# Enhancing Phenol Conversion Rates in Saline Anaerobic Membrane Bioreactor Using Acetate and Butyrate as Additional Carbon and Energy Sources

**DOI:** 10.3389/fmicb.2020.604173

**Published:** 2020-11-30

**Authors:** Víctor S. García Rea, Julian D. Muñoz Sierra, Laura M. Fonseca Aponte, Daniel Cerqueda-Garcia, Kiyan M. Quchani, Henri Spanjers, Jules B. van Lier

**Affiliations:** ^1^Sanitary Engineering Section, Department of Water Management, Delft University of Technology, Delft, Netherlands; ^2^KWR Water Research Institute, Nieuwegein, Netherlands; ^3^Institute of Ecology, National Autonomous University of Mexico, Mexico City, Mexico

**Keywords:** phenol, anaerobic membrane bioreactor, enhanced phenol conversion rate, acetate, butyrate, microbial community, *Syntrophorhabdus* sp.

## Abstract

Phenolic industrial wastewater, such as those from coal gasification, are considered a challenge for conventional anaerobic wastewater treatment systems because of its extreme characteristics such as presence of recalcitrant compounds, high toxicity, and salinity. However, anaerobic membrane bioreactors (AnMBRs) are considered of potential interest since they retain all micro-organism that are required for conversion of the complex organics. In this study, the degradation of phenol as main carbon and energy source (CES) in AnMBRs at high salinity (8.0 g Na^+^⋅L^–1^) was evaluated, as well as the effect of acetate and an acetate-butyrate mixture as additional CES on the specific phenol conversion rate and microbial community structure. Three different experiments in two lab-scale (6.5 L) AnMBRs (35°C) were conducted. The first reactor (R1) was fed with phenol as the main CES, the second reactor was fed with phenol and either acetate [2 g COD⋅L^–1^], or a 2:1 acetate-butyrate [2 g COD⋅L^–1^] mixture as additional CES. Results showed that phenol conversion could not be sustained when phenol was the sole CES. In contrast, when the reactor was fed with acetate or an acetate-butyrate mixture, specific phenol conversion rates of 115 and 210 mgPh⋅gVSS^–1^ d^–1^, were found, respectively. The syntrophic phenol degrader *Syntrophorhabdus* sp. and the acetoclastic methanogen *Methanosaeta* sp. were the dominant bacteria and archaea, respectively, with corresponding relative abundances of up to 63 and 26%. The findings showed that dosage of additional CES allowed the development of a highly active phenol-degrading biomass, potentially improving the treatment of industrial and chemical wastewaters.

## Introduction

Rapid industrialization has generated many industrial effluents that constitute a major source of pollution (Lin et al., 2013). Currently, many of these industrial effluents are successfully treated using anaerobic high-rate treatment processes (Van Lier et al., 2015). However, some industrial wastewaters represent a challenge for conventional anaerobic wastewater treatment systems. Wastewater characteristics that are considered extreme, such as high organic pollutant concentration, presence of recalcitrant or refractory as well as toxic or inhibitory compounds, and high salinity, reduce the performance of conventional anaerobic systems, which leads to process imbalance or reactor failure (Dereli et al., 2012). Dereli et al. (2012), proposed the use of anaerobic membrane bioreactors (AnMBR) to treat industrial wastewater with extreme characteristics, especially because of their effective biomass retention and the production of suspended-solids-free effluents, making them suitable for water reclamation.

Chemical and petrochemical wastewater, such as coal gasification, is an example of an industrial effluent with toxic phenolics as the major organic pollutants (Li et al., 2017). Nevertheless, other compounds such as acetate or butyrate, which could be used by microorganisms as carbon and energy sources (CES), are also present in coal gasification wastewater as common contaminants (Singer et al., 1978; Blum et al., 1986; Ji et al., 2016). Besides, it has been reported that coal-related industries wastewaters have a high concentration of total dissolved solids, ranging from 174 to 2,000 mg⋅L^–1^ (Maiti et al., 2019). Furthermore, under closed-water-loops, increasing salinity in the wastewater is expected.

Several studies have researched the anaerobic degradation of phenol, as well in some studies different CES have been used to promote or enhance the degradation of phenol under anaerobic conditions (Liang and Fang, 2010), e.g., glucose (Tay et al., 2001), volatile fatty acids (VFAs) (Carbajo et al., 2010), or acetate (Muñoz Sierra et al., 2017). Additional CES have been used as well during the reactor start-up, or for the degradation of mixtures of phenolic compounds. However, in most of these studies, it remains unclear how and to what extent these substrates promoted or increased phenol degradation.

We have identified four possible mechanisms that could explain the effect of additional CES on the degradation of toxic or inhibitory compounds: 1. Co-metabolism, a process that was initially defined as the catabolic degradation of a recalcitrant substrate without using the generated energy to promote or sustain cell growth (Horvath, 1972). In this process, the degradation of the non-easily degradable compound is dependent on the presence of a main substrate, or primary source, which is commonly an easily degradable compound (Horvath, 1972; Bertrand et al., 2015). However, (Wackett, 1996) attributed the enhancement in the conversion rate to an unknown or unidentified effect in the metabolic net between the different microbial populations, which are present in non-defined mixed cultures.

2. Direct usage of additional CES by the toxicant degraders to increase their metabolic capability (Kennes et al., 1997; Tay et al., 2001; Gali et al., 2006). Meaning that: a) additional CES could be used as a substrate to increase the anabolism of the degraders, promoting its growth, thus increasing its fraction in the biomass. b) Increasing the catabolic activity (i.e., uptake rate) of the degraders; or c) both processes are increased (Supplementary Material S1). This would imply, e.g., for phenol, that phenol degraders could use another (easily biodegradable) CES. The latter contrasts to the general comprehension that in anaerobic digestion (AD) process, specific (physiological) microbial populations have well-defined narrow substrate ranges (Batstone et al., 2002).

3. Effect of syntrophy on the thermodynamics of compound degradation. In the case of phenol conversion, a constant level of hydrogen consumption is required, which serves as an electron sink for favorable thermodynamics conditions (Qiu et al., 2008). Therefore, the development of a sound hydrogenotrophic methanogenic subpopulation is indispensable for establishing efficient anaerobic oxidation of aromatics and/or their degradation products.

4. An increase in intermediate compounds involved in the conversion of phenolics. Phenol degradation under anoxic conditions occurs via carboxylation of the phenolic ring to form 4-hydroxybenzoate (Schink et al., 2000; Gibson and Harwood, 2002; Fuchs et al., 2011). It has been reported that under strict anaerobic (methanogenic) conditions, phenol degradation is dependent on a sufficiently high CO_2_/HCO_3_^–^ and H_2_ concentration (Knoll and Winter, 1987; Karlsson et al., 1999; Fuchs, 2008). Veeresh et al. (2005) reported that dosage of additional CES increased the phenol-degrading activity of the biomass, hypothesizing that this was due to a higher phenol hydrogenation rate, resulting in a better cleavage of the phenolic ring.

In contrast to these four mechanisms, addition of an extra CES, such as acetate, has been shown to inhibit the anaerobic degradation of terephthalic acid (Kleerebezem, 1999.), which even though is not a phenolic compound, shares the same degradation pathway (under anoxic/anaerobic conditions) with phenol (Nobu et al., 2015). So, the effect of CESs’ dosage on the degradation of recalcitrant and toxic or inhibitory compounds is not fully understood. Moreover, there is few information regarding the effect of the dosage of CES on the specific phenol conversion rate (sPhCR), especially in AnMBRs under saline conditions.

As well, little is known regarding the microbial community structure and dynamics in AnMBRs under saline conditions treating toxic compounds such as phenol. Furthermore, no study has been conducted to determine how the microbial community is shifted by either the increase in the loading rates and/or the addition of extra CES, especially in suspended biomass systems as the one present in the AnMBR.

This study researched the effect of the addition of acetate or a mixture of acetate-butyrate as additional CES on the sPhCR of AnMBRs and on the microbial community related to the degradation process, with particular focus on the phenol degraders and the methanogens. The effect of phenol on the acetotrophic specific methanogenic activity (SMA) and the sPhCR in batch experiments were assessed as well.

## Materials and Methods

### Analytical Techniques

#### Chemical Oxygen Demand

During the operation of the reactors, COD in the feed and the permeate was measured using a spectrophotometer (DR3900, Hach Lange, Germany). Hach Lange Kits (Hach Lange, Germany) were used following the instructions of the manufacturer. Proper dilutions were done to avoid Cl^–^ interference.

#### Phenol, Volatile Fatty Acids, and Benzoate Concentrations

Phenol, VFAs, and benzoate were measured by a gas chromatograph (GC) (Agilent Technology) equipped with a flame ionization detector (FID) with a capillary column (type HP-PLOT/U) with a size of 25 m × 320 mm × 0.5 mm. Helium was used as carrier gas at a flow rate of 67 mL⋅min^–1^ and a split ratio of 25:1. The oven temperature was increased from 80 to 180°C in 10.5 min. Injector and detector temperatures were 80 and 240°C, respectively, and the injection volume was 1 μL.

For the preparation of the samples, approximately 1 mL of permeate was filtered through a 0.45 μm filter (Chromafil Xtra PES-45/25). Depending on the dilution required to have phenol, VFAs, and benzoate in the measurable range of the GC, a certain volume of the filtrate was mixed with pentanol (320 mg⋅L^–1^) to obtain a final volume of 1.5 mL. Finally, 10 μL of formic acid (95%) (Merck, Germany) were added to the vial. Phenol concentration was double-checked with a spectrophotometer (Merck, Germany) using Merck – Spectroquant Phenol cell kits (Merck, Germany) following manufacturers’ instructions.

### Batch Tests

#### Specific Methanogenic Activity Inhibition by Phenol

Batch tests with initial phenol concentrations of 50 (*n* = 2), 200 (*n* = 6), and 500 (*n* = 6) mg⋅L^–1^ were carried out in 250 mL Schott glass (Schott, Germany) reactors. Biomass samples (60–80 mL) were taken from the AnMBR to have a final volume of 200 mL at a VSS concentration of 4 g⋅L^–1^ (Inoculum/Substrate = 2 for the control with 2.0 gAc-COD⋅L^–1^). Macro- and micronutrients, phosphate buffer solutions, and Na^+^ as NaCl were dosed as specified in section “Experimental Setup and Reactors Operation.” A shaker (New Brunswick^TM^, Eppendorf, Germany) at 130 rpm and at 35°C was used for the incubation. Methane production was continuously measured by an AMPTS (Bioprocess Control, Sweden) following manufacturer’s instruction. The tests were stopped when the acetate was completely depleted after 48–72 h approximately.

#### Phenol Degradation Tests

Batch tests with a phenol concentration of 500 mg⋅L^–1^ (*n* = 5) were performed. The batch tests were conducted in 250 mL Schott (Schott, Germany) glass reactors. Biomass was taken from the AnMBR to have a final VSS concentration of 4 g⋅L^–1^. The batch reactors were supplemented with macro- and micronutrient solution, phosphate buffer solutions, and Na^+^ (as NaCl) as specified in section “Experimental Setup and Reactors Operation.” The bottles were incubated at 35°C and 130 rpm. Samples (1–2 mL) were periodically taken, and phenol concentration was measured.

### Experimental Setup and Reactors Operation

#### Anaerobic Membrane Bioreactor Setup

**FIGURE 1:**
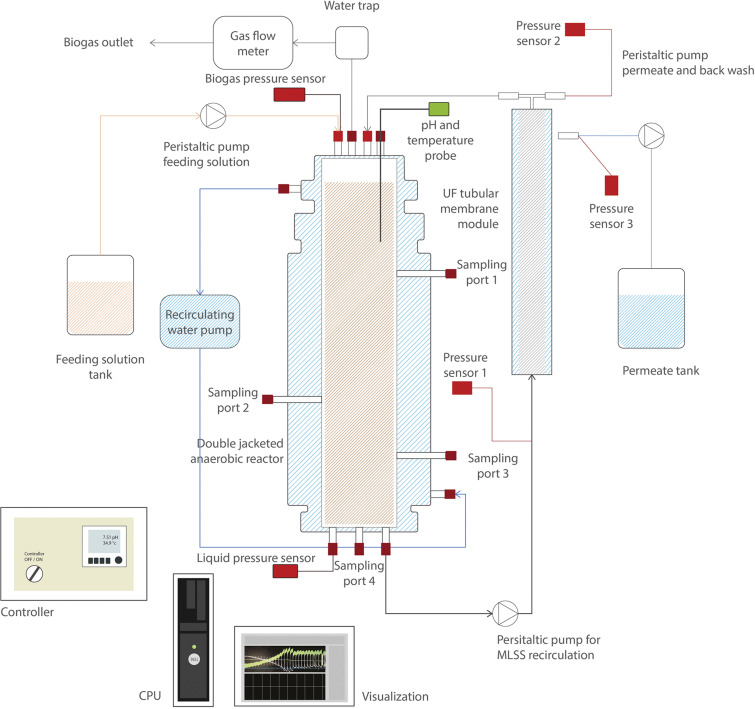
Scheme of the side-stream anaerobic membrane bioreactor (AnMBR) setup.

Two AnMBRs (6.5 L working volume) were used for the continuous experiment. Figure 1 depicts a scheme of the reactors’ setup. The temperature of the reactors was kept at 35.0 ± 1.0°C by a water bath (Tamson Instruments, Netherlands) circulating warm water through the reactor double-jacketed wall. Mixing in the reactor was ensured by internal sludge circulation with a reactor turnover of approximately 200 times⋅d^–1^. The setup used two peristaltic pumps (Watson Marlow 12 U/DV, 220 Du) for the feeding solution and permeate extraction, respectively, and one pump (Watson Marlow 620U) for the sludge recirculation. Temperature and pH were measured online by pH/temperature probes (Endress & Hauser Memosens and Mettler Toledo). The biogas production rate was measured by a gas meter counter MGC-1 PMMA (Ritter, Milligas, and MGC-10).

Each reactor was coupled to an external pressure-driven ultrafiltration (nominal pore size of 30 nm) PVDF membrane module (X-Flow, Pentair). Membranes were 64 cm length and 0.52 cm diameter, and were operated at a cross-flow velocity of 0.8 m⋅s^–1^ (*Q* ≈ 1,450 L⋅d^–1^). Reactors worked at a constant flux of 6 L⋅m^–2^⋅h^–1^. The transmembrane pressure (TMP) was measured by three different pressure sensors (AE Sensors, The Netherlands), with a range of −800 to 600 mbar, that were located at the entrance and outlet of the membrane, and at the permeate side. Reactor volume was controlled by two pressure sensors (AE Sensors, The Netherlands) with a range of 0 to 100 mbar, one was located on top of the reactor to measure the gas pressure, and the other at the bottom of the reactor, to measure the hydrostatic pressure plus the gas pressure.

Biomass was already acclimated to high sodium concentration, and phenol and acetate degradation (Muñoz Sierra et al., 2018). Before starting the continuous experiments, the biomass was mixed and then evenly distributed between the two reactors.

#### AnMBRs Operation and Model Wastewater Composition

In the first reactor (R1), phenol was targeted to be, besides the yeast extract, the sole CES. For the second reactor (R2), acetate and a 2:1 acetate-butyrate mixture were added as additional CES (Table 1). Besides the first 10 days of operation of R1 where the HRT was decreased from 7 to 4 d, the HRT in the AnMBRs was maintained at 4 d. The average SRT was calculated as SRT = X_*reactor*_*ave* [gVSS]/X_*removed*_[gVSS⋅d^–1^], where X_*removed*_ resulted from the biomass withdrawn for samplings divided by the days between each determination of solids. SRT values of 4,300 ± 1,600 (R1), 4,500 ± 1,700 d (R2a), and 5,300 + 2,040 d (R2b) were found.

**TABLE 1:** Influent concentration and loading rates of the different carbon sources during the operation of the AnMBRs.

*Reactor*	*Stages*	*Operation day*	*Phenol [g⋅L^–1^]*	*Phenol loading rate [mgPh⋅gVSS^–1^d^–1^]*	*Acetate [gCOD⋅L^–1^]*	*Acetate loading rate [gCOD-Ac⋅gVSS^–1^d^–1^]*	*Butyrate [gCOD⋅L^–1^]*	*Butyrate loading rate [gCOD-Bu⋅gVSS^–1^d^–1^]*
R1	I	0–59	0.5	10, 28, 42	4.7, 3.5, 1.2, 0.3, 0	100, 76, 25, 7, 0	N/A	N/A
	II	60–99	0.5, 0.9, 0.5	42, 52, 31	0	0	N/A	N/A
	III	100–115	0	0	2.5	54	N/A	N/A
R2 (a)	I	0–43	0.5	25	4.7, 3.5, 2.3, 2.0	236, 177, 113, 100	N/A	N/A
	II	44–100	0.5, 1.5, 3.0, 6.0, 8.2	25, 75, 115, 230, 317	2.0	100, 75	N/A	N/A
	III	101–115	0.0	0	9.1	350	N/A	N/A
R2 (b)	I	0–30	0.5	≈17	1.33	≈48	0.66	≈24
	II	31–110	0.75, 1.0, 1.5, 2.7, 6.5, 11.1	30, 42, 62, 108, 195, 265	1.33	≈48	0.66	≈24

For the model wastewater composition, and based on a modification to Hendriks et al. (2018) and Muñoz Sierra et al. (2018), per each gram of COD in the feeding solution, 1.5 mL of macronutrients solution, 0.76 mL of micronutrients solution, 2.2 mL of buffer phosphate solution A, 3.4 mL of buffer phosphate solution B, and 50 mg of yeast extract (Sigma Aldrich) were added, as well, to the feeding solution (Table 2). Enough NaCl was added to the feeding to keep a Na^+^ concentration of 8.0 g⋅L^−1^.

**TABLE 2:** Micro- and macro nutrient and buffer solutions dosed in the AnMBRs.

*Solution*	*Composition*
Micronutrient	EDTA-Na_2_ [1.0 g⋅L^–1^], H_3_BO_3_ [0.050 g⋅L^–1^], MnCl_2_⋅4H_2_O [0.50 g⋅L^–1^], FeCl_3_⋅6H_2_O [2.0 g⋅L^–1^], ZnCl_2_ [0.050 g⋅L^–1^], NiCl_2_⋅6H_2_O [0.050 g⋅L^–1^], CuCl_2_⋅2H_2_O [0.030 g⋅L^–1^], Na_2_SeO_3_ [0.10 g⋅L^–1^], (NH_4_)_6_Mo_7_O_2_⋅4H_2_O [0.090 g⋅L^–1^], Na_2_WO_4_ [0.080 g⋅L^–1^], and CoCl_2_⋅6H_2_O [2.0 g⋅L^–1^].
Macronutrient	NH_4_Cl [170 g⋅L^–1^], CaCl_2_⋅2H_2_O [8 g⋅L^–1^], and MgSO_4_⋅7H_2_O [9 g⋅L^–1^].
Buffer solution A	K_2_HPO_4_⋅3H_2_O [45.6 g⋅L^–1^].
Buffer solution B	NaH_2_PO_4_⋅2H_2_O [31.2 g⋅L^–1^].

### Microbial Community Analysis

#### DNA Extraction, Quantification, and Amplification

Biomass samples corresponding to 1.5–2.0 mL of MLSS were regularly taken from the AnMBRs during the operation of the reactors. The biomass was transferred to Eppendorf tubes (Eppendorf, Germany) and centrifuged in a microcentrifuge (Eppendorf, Germany) at 10,000 *g* for 5 min. The supernatant was discarded and the biomass pellets were frozen and stored at −80°C. For the DNA extraction, the biomass pellet were thawed, and the DNA was extracted with the DNeasy UltraClean Microbial Kit (Qiagen, Germany). Qubit3.0 DNA detection (Qubit dsDNA HS assay kit, Life Technologies, United States) was used to verify DNA quality and quantity.

DNA (16S rRNA gene) amplification was done by Illumina Novaseq 6000 platform by Novogene. The hypervariable regions V3–V4 were amplified using the primer set 341F [(5′–3′) CCTAYGGGRBGCASCAG] and 806R [(5′–3′) GGACTACNNGGGTATCTAAT]. The PCR reactions were carried out with Phusion High- Fidelity PCR Master Mix (New England Biolabs).

#### DNA Data Processing

The paired-end reads (2 × 250) were processed in the QIIME2 pipeline (Bolyen et al., 2019). After manual inspection, the forward and reverse reads were truncated in the position 250 in the 3′ end, and the forward reads were trimmed in the position 35 in the 5′ end. Then, the reads were denoised, and the amplicon sequences variants were resolved with the DADA2 plugin (Callahan et al., 2016) removing chimeric sequences with the “consensus” method. The taxonomy of the representative sequences of the amplicon sequences variants was assigned with the classify-consensus-vsearch plugin (Rognes et al., 2016), using the SILVA 132 database (Quast et al., 2013) as reference. The feature table was exported to the R environment to perform the statistical analysis with the phyloseq library (Mcmurdie and Holmes, 2013). The sequences were deposited in the SRA (NCBI) database under the accession number PRJNA663299.

#### Canonical Correspondence Analysis

A canonical correspondence analysis (CCA) was calculated, using the Weighted Unifrac distance metric and the specific phenol loading rate (sPhLR) and sPhCR as explanatory variables. The statistical significance of the ordination in the CCA was tested with an ANOVA at a *p*-value <0.05.

## Results and Discussion

### Acetoclastic SMA Inhibition by Phenol, and Butyrate Degradation Tests

**FIGURE 2:**
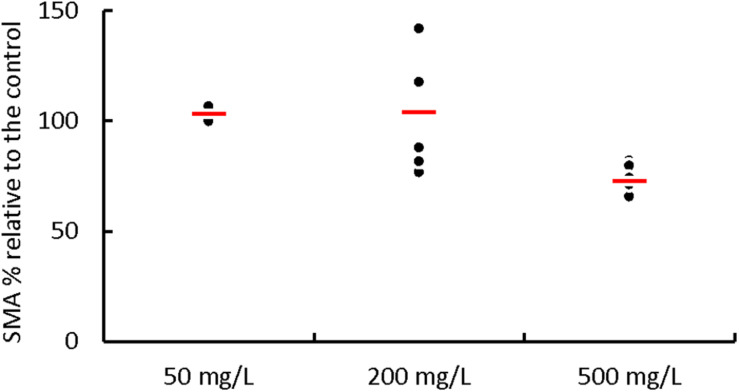
Effect of the phenol addition on the percentage of the acetoclastic SMA of the AnMBR sludge. Each point represents the percentage of the SMA value when phenol, at different concentrations, was added in comparison to the value of the control SMA (acetate at 2 g COD⋅L^–1^). The thick red line represents the average SMA value for the different phenol concentrations.

Batch tests with initial phenol concentrations of 50, 200, and 500 mg⋅L^–1^ were performed to determine a possible inhibition by phenol on the acetoclastic SMA of the AnMBR biomass (Figure 2) (Supplementary Material S2, Supplementary Table S1). At initial phenol concentrations of 50 and 200 mg⋅L^–1^, the SMA values were 3.4 ± 4.8% and 4.3 ± 23.6%, respectively, higher than the control (acetate 2.0 g COD⋅L^–1^), meaning that at these concentrations, there was no inhibition in the acetoclastic methanogens due to phenol. On the other hand, the SMA at an initial phenol concentration of 500 mg⋅L^–1^ was 27.0 ± 6.6% lower than the control SMA, meaning that the methanogens were inhibited by phenol.

AD inhibition by phenol is a phenomenon reported in literature (Olguin-Lora et al., 2003; Liang and Fang, 2010). Although phenol may affect all main physiological microbial populations of the AD process, it is reported, as with other inhibitory or toxic substances (Astals et al., 2015), that the acetoclastic methanogenic population could be the most affected group (Chen et al., 2008). Half maximum inhibitory concentration (IC_50_) ranges between 50 and 1,750 mg⋅L^–1^ for non-adapted and phenol-degrading biomass have been reported (Fang and Chan, 1997; Olguin-Lora et al., 2003; Collins et al., 2005; Muñoz Sierra et al., 2017).

Furthermore, we performed all batch experiments at 8.0 g Na^+^⋅L^–1^ which might have had an impact as well, even though the biomass was adapted to this Na^+^ concentration. In agreement with literature (Fang and Chan, 1997; Olguin-Lora et al., 2003; Collins et al., 2005; Muñoz Sierra et al., 2017), the results obtained in this research showed that a phenol concentration of 50 mg⋅L^–1^ caused no negative effect on the SMA when compared to the control without phenol. As well, the average SMA at phenol concentration of 200 mg⋅L^–1^ was not affected, but phenol concentration of 500 mg⋅L^–1^ decreased the SMA with 27%. Butyrate at a concentration up to 3.0 g COD⋅L^–1^ did not cause inhibition problems (Supplementary Material S3).

### Phenol Degradation in Batch Assays

**FIGURE 3:**
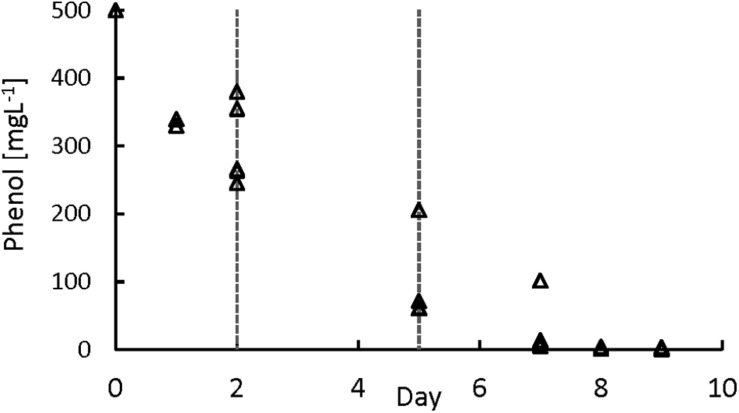
Phenol degradation batch assays. The lines represent the period used for the determination of the specific phenol conversion rate.

To further study the phenol degradation kinetics, a series of batch tests with phenol concentrations of 500 mgPh⋅L^–1^ were performed (Figure 3). For calculating the sPhCR, the period between day 2 and day 5 of the assays was chosen to get the part of the curve that avoids a possible inhibition of the phenol degraders by high phenol concentration (Supplementary Material S1). The average sPhCRs calculated during the batch test were 17.8 ± 2.6 mgPh⋅gVSS^–1^ d^–1^.

Several sPhCR values for granular biomass have been reported in batch tests, such as 126 (Tay et al., 2000), 65 (Razo-Flores et al., 2003), and 38–93 (Fang et al., 2004) mgPh⋅gVSS^–1^ d^–1^ which are higher than the average value of 16.6 ± 1.9 mgPh⋅gVSS^–1^ d^–1^ that we obtained. However, in our study, the sPhCRs assessed in the batch tests were lower than the sPhCRs determined in the continuous experiment (section “AnMBR Operation”).

### AnMBR Operation

#### AnMBR Operation Toward Phenol as the Main Carbon and Energy Source

**FIGURE 4:**
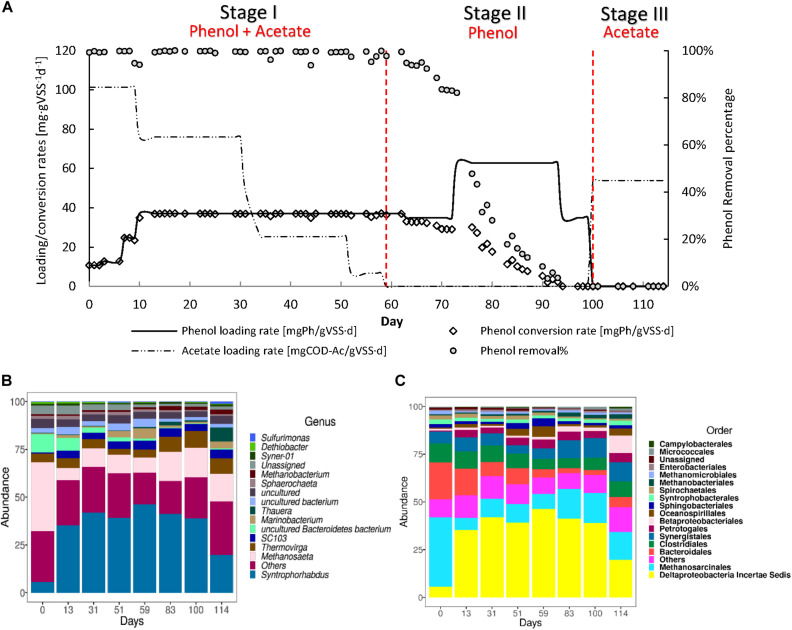
Operation **(A)** and microbial community dynamics **(B,C)** of the R1 toward the usage of phenol as the main carbon and energy source (CES). The graph in **(A)** shows the phenol loading and conversion rates, the acetate loading rate, and the phenol removal percentage during the AnMBR operation. **(B,C)** shows the microbial community dynamics, as the more abundant microbial order **(B)** or genus **(C)** during the different operational days.

R1 was operated to assess whether phenol could serve as the sole CES (Figure 4A) and the maximum sPhCR that could be achieved. During stage I, in which acetate was stepwise decreased and the sPhLR was stepwise increased in ten days from 12 to 42 mgPh⋅gVSS^–1^ d^–1^. During this period, the sPhCR remained the same as the sPhLR, i.e., 42 mgPh⋅gVSS^–1^ d^–1^, corresponding to a phenol removal efficiency exceeding 99%. In stage II, after the exclusion of acetate from the feeding, the sPhCR decreased to 29 mgPh⋅gVSS^–1^ d^–1^ during the days 59–72. When the phenol loading was increased to 62 mgPh⋅gVSS^–1^ d^–1^, the sPhCR and the removal percentage started to decrease, and on day 94, the sPhCR and the phenol removal efficiency were 0. During stage III, phenol was excluded from the influent because no phenol conversion was observed, and further intoxication of the reactor biomass was unwanted. Hence, the COD was replaced with acetate.

In our present study, it was not possible to achieve long-time AnMBR operation with phenol as the sole CES. This was confirmed with a second experiment (Supplementary Material S4, Supplementary Figure S1). Possibly, this inability might be attributed to the applied sodium concentration of 8 g⋅L^–1^, which has been hypothesized to decrease the phenol conversion (Wang et al., 2017). Under saline conditions, more of the catabolically generated energy from the substrate conversion, in this case phenol, will be spent on regulating a higher maintenance energy in the biomass, because of the increased osmotic pressure (Russell and Cook, 1995; He et al., 2017). A maximum sPhCR of 40 mgPh⋅gVSS^–1^ L^–1^ was determined from days 49 to 62 (Stage I) when phenol was the main CES, representing 80% of the total COD while acetate contributed to 20% of the COD. However, this sPhCR could not be sustained for more than five days after the acetate was excluded from the influent (Stage II).

For continuous reactor operation, anaerobic degradation of phenol as sole CES at different sPhCRs (6–690 mgPh⋅gVSS^–1^d^–1^) has been reported (Fang et al., 1996; Kennes et al., 1997; Zhou and Fang, 1997; Razo-Flores et al., 2003; Ramakrishnan and Gupta, 2008; Liang and Fang, 2010) (Table 3). Most of these studies refer to granular-sludge-based reactors, such as upflow anaerobic sludge blanket (UASB) reactors, under non-saline conditions. As an exception, Suidan et al. (1988), reported the successful continuous operation of a chemostat (suspended biomass) with phenol as the only CES.

**TABLE 3:** Studies dealing with phenol degradation either as main/unique carbon and energy source or with the dosage of additional carbon and energy sources.

Substrate [mg⋅L^–1^]	Sludge PhLR [gPh⋅gVSS^–1^ d^–1^]	Total Removal	sCH_4_ rate [L⋅gVSS^–1^ d^–1^]	CH_4_ yield [LCH_4_⋅gCOD^–1^]	gCH_4_-COD⋅gCOD^–1^	HRT	Vol [L]	Reactor type	References
**Phenol degradation with no additional carbon and energy source**									
Phenol (400)		90%		0.21	*53%*	0.14 d	0.66	rUASB	Chang et al. (1995)
Phenol (420–1,260)	*0.03-0.09*	98%	*0.075*	*0.35*	*89%*	12 h	2.8	rUASB	Fang et al. (1996)
Phenol (2,000)After rec (250 to 500)	*0.58*	99%	*0.001*	*0.47*	*117%*	6 h	35	rUASB	Lay and Cheng (1998)
Phenolics (600)	0.0182	30%	*0.001*	*0.02*	*5%*	24 h	7	UASB	Wang et al. (2010)
Phenol (50–700)	*0.024*	85%	*0.024*	0.415	*69%*	24 h	2.8	AFBR	De Amorim et al. (2015)
**Additional carbon and energy source dosage for reactor start-up or biomass reactivation**									
Phenol (234)	*0.006*	*92%*	*0.008*	0.338	*86%*	48 h	13.4	rAF	Li et al. (2016)
Phenol (1,260)	*0.26*	94%		0.308	*80%*	12 h	2.8	UASB	Fang et al. (2004)
Phenol (1,260)	*0.315*	86%	*0.304*	0.284	*72%*	12 h	2	UASB	Tay et al. (2000)
**Phenol degradation with additional carbon and energy source dosage**									
Phenol (105–1,260) + Glucose	*0.06–0.28*	98%				12 h	2	UASB	Tay et al. (2001)
Phenol (625)+ acetate (3,850)+ Na (10 g L^–1^)	0.1042	100%				48 h	3.5	rUASB-AF	Wang et al. (2017)
Phenol (1,000)+ Acetate (1,000)		99%		0.39	*100%*	5–2.5 h	10	GAC-AFBR	Lao (2002)
Phenol (500–1,000) + VFAs	0.07–0.14	98%		0.32	*87%*	12 h	3.5	rEGSB-AF	Scully et al. (2006)
Phenol (672)+ VFAs		95%		0.28	*72%*	0.43 d	1.8	AFBR	Carbajo et al. (2010)
Phenol (500) + Acetate + NaCl [4.7–20 g Na^+^⋅L^–1^]	0.012	99%				6.5 d	6.5	AnMBR	Muñoz Sierra et al. (2018)
*Phenol (500)* + *NaCl (8 gNa^+^⋅L^−1^)*	*0.042*	*97%*	*0.036*	*–*	*–*	*4 d*	*6*	*AnMBR*	*This study*
*Phenol (3,000)*+ *Acetate (2 gCOD⋅L^−1^)* + *NaCl (8 gNa^+^⋅L^−1^)*	*0.115*	*100%*	*0.114*	*0.27*	*68%*	*4 d*	*6*	*AnMBR*	*This study*
*Phenol (6,500)*+ *Acetate-Butyrate (2:1) (2 gCOD⋅L^−1^)* + *NaCl (8 gNa^+^⋅L^−1^)*	*0.200*	*100%*	*–*	*–*	*–*	*4 d*	*6*	*AnMBR*	*This study*

An anaerobic granule consists of several populations of microorganisms forming an ecosystem, in which the products of a specific population serves as the substrate for others in a very close vicinity. Moreover, methanogens and phenol-degraders in the inside are only exposed to very low phenol concentrations when phenol is indeed readily degraded in the continuous system. Subsequent phenol conversion in the granule interior provides the conversion intermediates as a substrate for the other populations, allowing the use of phenol as the sole CES. In suspended biomass systems, such as an AnMBR, the phenol concentration is the same for all biomass, while distances between microbial species are much larger, making these systems much more sensitive to increased phenol concentrations.

#### Effect of the Addition of Acetate on the Specific Phenol Conversion Rate

**FIGURE 5:**
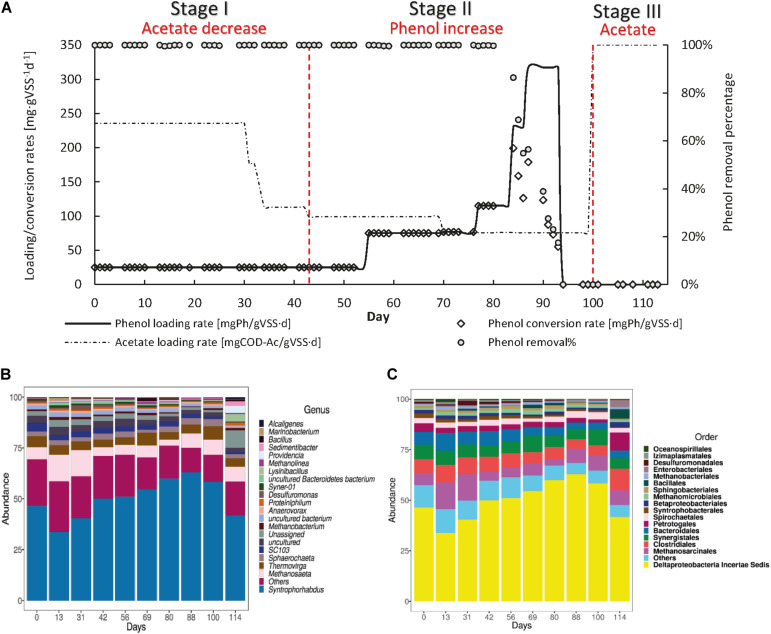
Operation **(A)** and microbial community dynamics **(B)** of the R2(a) with acetate [2 g COD ⋅L^–1^] as an additional carbon and energy source. The graph in **(A)** shows the phenol loading and conversion rates, the acetate loading rate, and the phenol removal percentage during the AnMBR operation. **(B,C)** show the microbial community dynamics, as the more abundant microbial order **(B)** or genus **(C)** during the different operational days.

R2(a) was operated to determine the effect of the addition of acetate as an extra CES on the sPhCR (Figure 5A). In stage I, the acetate-COD concentration in the influent was decreased from 4.7 to 2.0 gCOD L^–1^, corresponding to an acetate loading rate of 100 mgAc-COD⋅gVSS^–1^ d^–1^, while the sPhLR was maintained at 25 mgPh⋅gVSS^–1^ d^–1^, corresponding to a phenol concentration in the influent of 0.5 gPh⋅L^–1^. During this stage, the sPhCR was 25 mgPh⋅gVSS^–1^ d^–1^, corresponding to a phenol removal percentage of ≈100%.

In stage II, the sPhLR was stepwise increased from 75 to 230 mgPh⋅gVSS^–1^ d^–1^, corresponding to phenol concentrations in the influent of 1.5 and 8.2 g⋅L^–1^, respectively. Phenol removal of 100% was observed with phenol loading rates of 75 and 115 mgPh⋅gVSS^–1^ d^–1^. At a loading rate of 230 mgPh⋅gVSS^–1^ d^–1^, the sPhCR and the removal efficiency decreased to 86 mgPhg⋅VSS^–1^ d^–1^ and 55%, respectively. The further increase in the sPhLR (320 mgPh⋅gVSS^–1^ d^–1^) on day 87 caused an intoxication of the AnMBR, which was observed as a decreased sPhCR that was eventually halted. During days 94 to 100, phenol in the feeding solution was excluded and acetate concentration was kept at 2.0 g COD⋅L^–1^. In stage III, the reactor was fed with only acetate at a concentration of 9.1 g COD⋅L^–1^ to avoid further intoxication of the biomass.

Acetate has been used as an additional CES in the process of biological phenol degradation under anaerobic conditions, either during the reactor start-up (Razo-Flores et al., 2003) or operation (Wang et al., 2017; Muñoz Sierra et al., 2018, 2019) (Table 3). Wang et al. (2017) reported UASB reactors treating synthetic wastewater with acetate concentration of 3.6 g COD⋅L^–1^ and phenol concentrations ranging from 0.1 to 2.0 g⋅L^–1^, operating under saline conditions with Na^+^ concentration of 10 g⋅L^–1^. They reported maximum sPhCR of 20 and 13 mgPh⋅gVSS^–1^ d^–1^ for batch and continuous reactors, respectively. Working with AnMBRs, Muñoz Sierra et al. (2019), reported maximum sPhCRs of 87 mgPh⋅gVSS^–1^ d^–1^ at a sodium concentration of 18 g⋅L^–1^, corresponding to concentrations in the influent of 5 g Phenol⋅L^–1^ and an acetate concentration of approximately 30 g COD⋅L^–1^. For comparison, in this experiment, we found a maximum stable sPhCR of 115 mgPh⋅gVSS^–1^ L^–1^ when acetate [2 g COD⋅L^−1^] was used as additional CES.

#### Effect of the Addition of the Acetate-Butyrate on the Specific Phenol Conversion Rate

**FIGURE 6:**
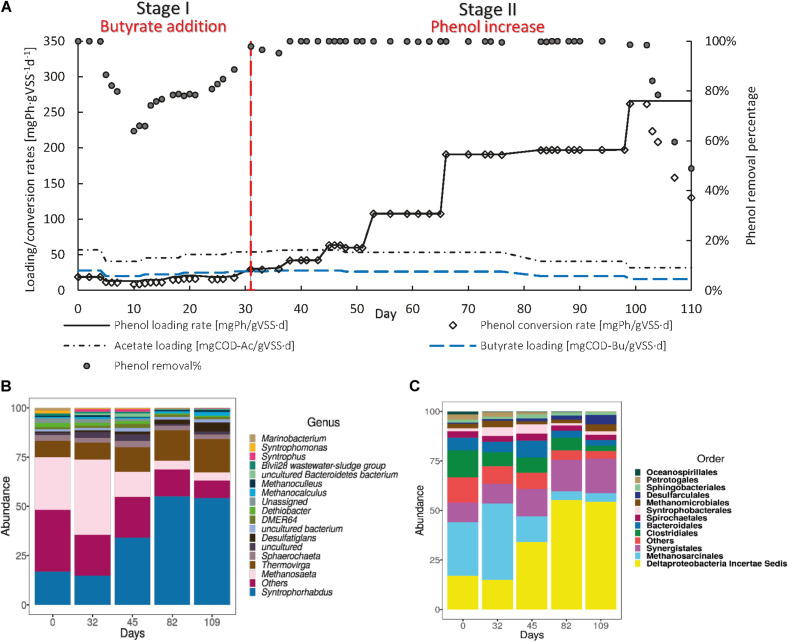
Operation **(A)** and microbial community dynamics **(B)** of the R2(b) with a 2 g COD⋅L^–1^ 2:1 acetate-butyrate mixture as an additional carbon and energy source. The graph in **(A)** shows the phenol loading and conversion rates, the acetate and butyrate loading rates, and the phenol removal percentage during the AnMBR operation. **(B,C)** show the microbial community dynamics, as the more abundant microbial order **(B)** or genus **(C)** during the different operational days.

After the recovery of the biomass from phenol intoxication, R2 was fed with a 2:1 acetate-butyrate mixture at a concentration of 2 g COD⋅L^–1^ to determine the effect of the dosage of an additional CES that intrinsically generates H_2_ during its conversion on the sPhCR and the phenol degraders and methanogens (Figure 6A). During stage I, the sPhLR was kept at an average value of 17.1 ± 1.31 mgPh⋅gVSS^–1^ d^–1^, corresponding to an average phenol concentration in the influent of 0.5 g⋅L^–1^. On day 10 after the dosage of butyrate, the sPhCR and the phenol removal efficiency decreased to 8.4 mgPh⋅gVSS^–1^ d^–1^ and 64%, respectively. Possibly, the increased butyrate concentration impacted the phenol conversion pathway. Nobu et al. (2017) indicated that phenol converting microorganisms such as *Syntrophorhabdus* sp., have an alternative phenol degradation pathway, which yields one molecule of butyrate and one of acetate. Therefore, the increased butyrate concentration in the AnMBR could have decreased phenol degradation by product inhibition (Figure 6A).

During stage II, from day 32 to 98, a phenol removal efficiency of 100% was found up to a sPhLR (and sPhCR) of 200 mgPh⋅gVSS^–1^ d^–1^ (influent concentration 6.5 gPh⋅L^–1^), being amongst the highest anaerobic sPhCRs’ reported in the literature, and the highest value reported for suspended biomass under anaerobic and saline conditions (Table 3). When the sPhLR was increased to 265 mgPh⋅gVSS^–1^ d^–1^ (phenol concentration in the influent = 11.1 g⋅L^–1^), on day 99, the sPhCR and the phenol removal efficiency started to decrease. On day 110 the sPhCR and the removal efficiency had already decreased to 130 mgPh⋅gVSS^–1^ d^–1^ and 45%, respectively, meaning a reactor failure due to biomass intoxication.

### Analysis of the Microbial Community Dynamics During the Operation of the Reactors

#### Microbial Community Dynamics in the AnMBR Toward Phenol as the Main Carbon and Energy Source

To get an insight into the microbial community structure in the AnMBR biomass during the reactor operation and to determine what was the effect of the sPhLRs on this structure, we analyzed the V3–V4 regions of the 16S rRNA gene of several biomass samples. The analysis showed that during the whole reactor operation, the more abundant microorganisms at genus level, were the Deltaproteobacteria *Syntrophorhabdus* sp. (Figures 4B,C), which is a reported anaerobic syntrophic phenol degrader (Qiu et al., 2008; Nobu et al., 2015; Franchi et al., 2018), and the acetoclastic methanogen *Methanosaeta* sp. Together, these microorganisms represented ≈50% of the total microbial community; remarkably, no other genus had a relative abundance higher than 6% (Supplementary Material). However, other microorganisms such as *Thermovirga* sp. (5.8 ± 2.1%), *Marinobacterium* (2.1 ± 1.8%) (Figure 4C), and *Thauera* (1.5 ± 2.5%) were constantly present during the different stages. In this regard, *Thermovirga* sp., belonging to the order Clostridiales, has been reported in the microbial community of saline matrixes with either petrochemical (Dahle and Birkeland, 2006) or phenolic compounds (Wang et al., 2020). However, there are no studies suggesting phenol-degrading activity by this microorganism. *Marinobacterium* sp. has been reported as well as a community member of phenol-degrading reactors (Muñoz Sierra et al., 2019); although, most of the members of this genus are strictly aerobic, whereas *M. zhoushanense* is reported as facultative and halophilic microorganism (Han et al., 2016). *Thauera* sp. is a known phenol degrader, however, it has been reported as nitrate reducer.

During stage I, at day 0, the relative percentage of *Syntrophorhabdus* sp. was 5.6%, which increased to a maximum of 46.3% in the last period of this first stage (day 59) and had an average relative abundance of 40.7 ± 4.6%. For the methanogens, *Methanosaeta* sp. started at a relative abundance of 36.2%, and during the stage, it remained at an average of 8.4 ± 1.7%.

In stage II, the relative abundance of *Syntrophorhabdus* sp. decreased to 41.2% (day 83) and 38.9% (day 100), which seemed to correlate with the decrease in the sPhCR (section “Canonical Correspondence Analysis”) and therefore, the removal percentage. The acetoclastic methanogen *Methanosaeta* sp. remained as the most abundant methanogenic microorganism, with an average relative abundance of 15% (days 83 and 100).

During stage III corresponding to the intoxication period (section “AnMBR Operation Toward Phenol as the Main Carbon and Energy Source”), the relative abundance of *Syntrophorhabdus* sp. kept decreasing with respect to the previous stage to a value of 19.7%, while the methanogen *Methanosaeta* sp. had a relative abundance of 14.3%. For this stage, the low abundance of *Syntrophorhabdus* sp. coincided with the observed toxic effect of phenol and the fact that no more phenol but only acetate was present in the influent.

#### Microbial Community Dynamics in the AnMBR With Acetate as Additional Carbon and Energy Source

To determine the effect of the increase in the sPhLR and the dosage of acetate as additional CES on the microbial community structure, with a focus on the reported phenol degraders and the methanogens, we analyzed the microbial community structure of the R2(a) during different stages of its operation. We found, similar to R1, that the most abundant bacteria and archaea were *Syntrophorhabdus* sp. and *Methanosaeta*, respectively (Figures 5B,C). Same as in the operation of R1, no other genus had a relative abundance higher than 5%; although, *Thermovirga* sp. (4.7 ± 1.0%), was the next genus regarding relative abundance.

During stage I, the most abundant microorganism was the phenol degrader *Syntrophorhabdus* sp. with an average relative abundance of 40.2 ± 6.4%. The methanogens were mainly represented by *Methanosaeta* with an average abundance of 10.6 ± 4.1%. During stage II, after the sPhLR was increased, the relative abundance of *Syntrophorhabdus* sp. was increased, as well, to a maximum of 62.9% on day 88. However, on day 100, a decrease to 58.3% was observed, which correlated with the decrease in the phenol removal percentage (section “Canonical Correspondence Analysis”). For the methanogens, *Methanosaeta* sp. was the main microorganism during this stage with a maximum relative abundance of 7.5%.

During stage III, on day 114, a further decrease in the relative abundance of *Syntrophorhabdus* sp. to 41.8% was observed. However, the methanogen *Methanosaeta* sp. remained at a similar relative abundance as during stage II.

#### Microbial Community Dynamics in the AnMBR With the Acetate-Butyrate Mixture

To determine the effect of the sPhLR and an hydrogen-generating additional CES on the microbial community structure, with a focus on the reported phenol degraders and the methanogens, we analyzed the microbial community structure of the R2(b) during different stages of its operation (Figures 6B,C). For this reactor, the community was, again, mostly represented by the phenol degrader *Syntrophorhabdus* sp. and the acetoclastic *Methanosaeta* (>50%). As it was found in R1 and R2(a), there were no other genera with more than (5%) of relative abundance. However, in this reactor, the average relative abundance of *Thermovirga* sp. was 12.3 ± 3.9%, which suggests that this microorganisms could potentially have a role in the phenol degradation process.

At the beginning of stage I, *Syntrophorhabdus* sp. and *Methanosaeta* sp. represented 17.0 and 32.5% of the microbial community, respectively. On day 32, after the start of the butyrate dosage, and the observed decrease in the phenol removal percentage, there was a slight decrease in *Syntrophorhabdus* sp. to 14.9%; that, as discussed in section “Discussion on the Possible Phenol-Degrading-Enhancing Mechanisms,” it could possibly be related to an adverse effect of butyrate on the phenol degraders.

During stage II, on day 82, the relative abundance of *Syntrophorhabdus* sp. reached a maximum of 55.2%, which was 7% lower compared to the highest relative abundance of this bacteria when the maximum sPhCR in the reactor operation with acetate as additional CES was reached. Nonetheless, the sPhCR achieved with the acetate-butyrate mixture was 73% higher than that with only acetate (115 mgPh⋅gVSS^–1^ d^–1^). For the methanogens, the acetoclastic *Methanosaeta* sp. remained as the main microorganism. On day 109, the relative abundance of *Syntrophorhabdus* sp. remained at 54%.

#### Canonical Correspondence Analysis

**FIGURE 7:**
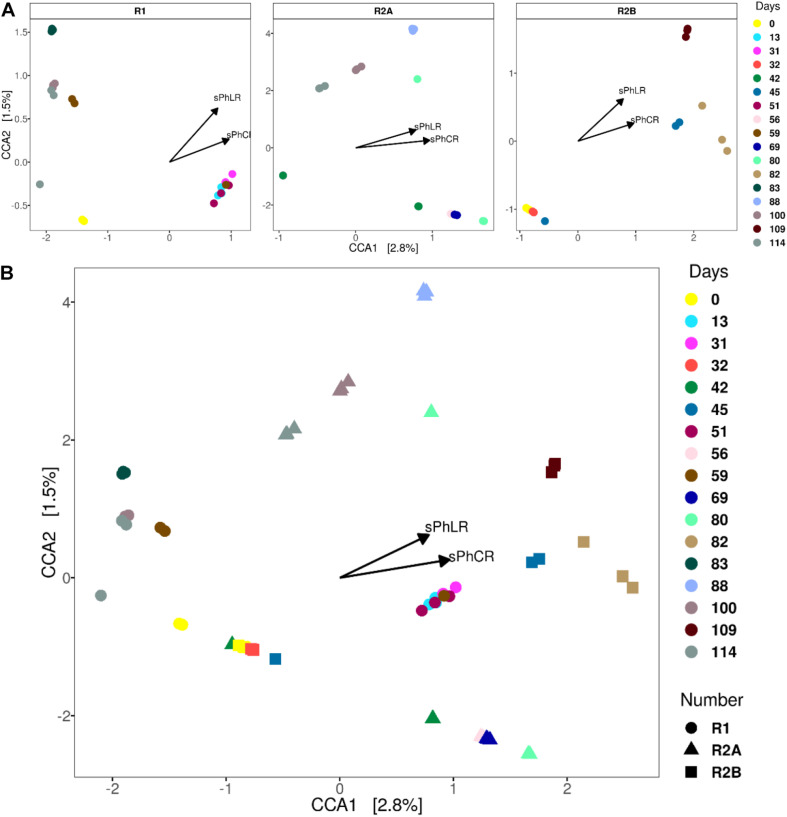
CCAs’ for each of the reactors **(A)** and for all three **(B)**. The samples that correspond to the reactors’ operation with increased loading and conversion rates group to right of the biplots.

Canonical (or constrained) correspondence analyses (CCA) were performed to assess the changes in the microbial community structure during the operation of the reactors; and therefore, to correlate the effect of the increase in the sPhLR, and the dosage of acetate or the acetate-butyrate mixture with the different microbial communities in R1, R2(a), and R2(b) (Figure 7). CCA is an ordination technique that recovers the response of the community structure to different physical environmental variables, in this case the sPhLR and the sPhCR (Palmer, 1993).

The biplots for each reactor (Figure 7A), in which the vectors indicate the importance of either sPhLR and sPhCR in the ordination process, showed a significant correlation (*p* < 0.05) between the community structures of the biomass of the reactors and the two variables. As it is seen in Figures 7A,B, the community structures at different days (R1 = 13, 31, 51; R2(a) = 56, 69 and 80; R2(b) = 45, 82, 109), corresponding to the points at the right of the biplot, were correlated with a higher sPhLR and sPhCR. In the comparison of the three reactors (Figure 7B), it was noticed that the samples of R2(a) and R2(b) did not group together; however, because of the position of each sample respecting the vectors (sPhLR and sPhCR), it can be concluded that those community structures were more related to higher sPhLR and sPhCR. Regarding these two vectors explaining the environmental variables, it was decided to use both sPhLR and sPhCR, because even though sPhLR was the independent variable, sPhCR represented the biological activity of the biomass.

### Discussion on the Possible Phenol-Degrading-Enhancing Mechanisms

Regarding to the four discussed mechanisms which could enhance the sPhCR, co-metabolism (1) and/or (2) direct usage of acetate as a catabolic substrate by the phenol degraders seems the less likely. As it has been reported, the phenol degrader *Syntrophorhabdus* sp., excretes acetate through a cation-acetate symporter (Nobu et al., 2017). Furthermore, phenol degraders are a physiological population with defined substrates (Eq. S6) (Qiu et al., 2008), being in this case, phenol and not acetate. However, the microbial community analyses results clearly indicate that with an increase in the sPhLR, the percentage of the biomass corresponding to the (reported) phenol degraders increased, reaching values over 50% of the total relative abundance, either at the order and genus levels (*Syntrophorhabdus* sp.), of the biomass of R2(a) and R2(b) (Figures 5, 6B,C). Note that the microbial community analyses were based on the study of the 16S rRNA gene, which may introduce inherent biases (Campanaro et al., 2018). As well, this analysis does not offer information about the metabolic state of the microorganisms, therefore, presence does not mean activity. Even though, with the addition of acetate or butyrate, the relative abundance of *Syntrophorhabdus* sp. reached values higher than 50%, which coincided with the highest sPhCRs achieved by the reactor. This correlation of the microbial community structure with the enhanced conversion was statistically confirmed by the CCA analysis (section “Canonical Correspondence Analysis”).

**FIGURE 8:**
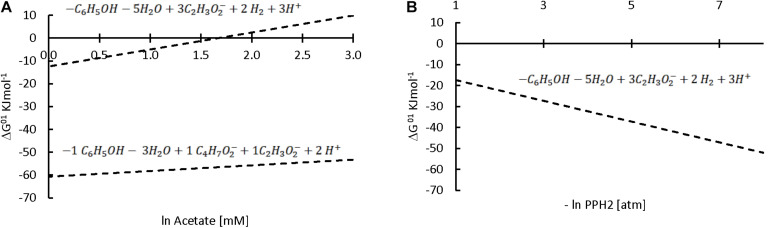
Effect of acetate (upper line) or butyrate (lower line) concentrations **(A)** or hydrogen partial pressure **(B)** on the ΔG^01^ of the anaerobic phenol degradation reaction. The stoichiometry of each reaction is shown above the corresponding line. Note that the *x* axis on **(A)** is ln while in **(B)** is –ln; therefore, higher concentrations, but lower partial pressures are expected at the right of *x* axis.

**FIGURE 9:**
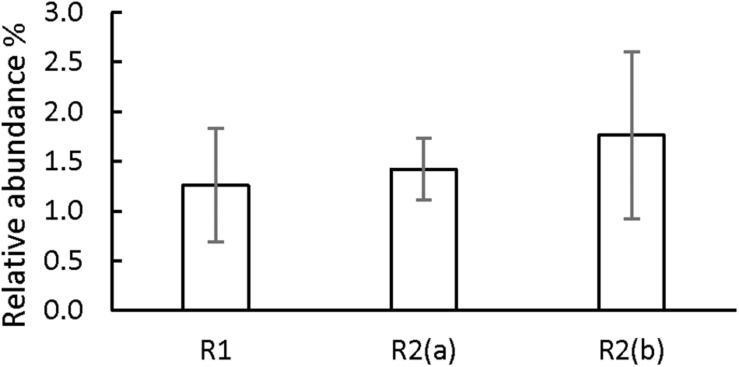
Average relative abundance percentage of the hydrogenotrophic methanogens in the operation of the three AnMBRs. *n* = 5 to 10, bars CI 95%.

On the other hand, our hypothesis is that the third identified mechanism, syntrophic association, could have played a role in the increase in sPhCR. Two different syntrophic associations with the methanogens are considered, either with the acetoclastics, which were the main methanogens (section “Analysis of the Microbial Community Dynamics During the Operation of the Reactors”) and with the hydrogenotrophic. Considering the acetoclastic methanogens, Figure 8 shows the effect of the concentration of acetate on the Gibbs free energy change (ΔG^01^, standard conditions and pH = 7) of phenol degradation under anaerobic conditions (section “Discussion on the Possible Phenol-Degrading-Enhancing Mechanisms,” thermodynamic data Supplementary Material S2, Supplementary Table S2), which shows that a low acetate concentration makes the reaction thermodynamically more favorable. Therefore, an abundant phenol-adapted acetoclastic methanogen population could offer advantages over phenol degradation. Nevertheless, R1 had similar values of the relative abundance of the acetoclastic methanogens (Supplementary Data Sheet S1). Regarding reactor R2(b) and the second syntrophic process, it has been shown that anaerobic mineralization of phenol requires the presence of balanced (syntrophic) associations that guarantee efficient interspecies electron transfer. In these associations, hydrogen-consuming microorganisms, such as the hydrogenotrophic methanogens, may act as the required electron sink (Qiu et al., 2008). For this reason, butyrate was added as additional CES in AnMBR experiment R2(b). However, the reactor fed with butyrate did not show a higher relative abundance of hydrogenotrophic archaea in comparison to the operation of the other reactors (Figure 9). Hydrogenotrophic microorganisms such as *Methanoculleus* sp. and *Methanocalculus* sp. were present at an average of 1.76 ± 0.68%, whereas, when only acetate was dosed as additional CES, hydrogenotrophic methanogens such as *Methanobacterium* sp. and *Methanolinea* sp. were found in similar relative abundances (1.42 ± 0.42%). A similar percentage of 1.26 ± 0.68% was found for the hydrogenotrophic *Methanobacterium* in the operation of R1, in which phenol as the sole CES was investigated.

Therefore, we hypothesize that the development of the (acetoclastic) methanogenic population could have enhanced the phenol conversion by keeping the degradation products, e.g., acetate, in a low concentration, allowing the constant conversion of phenol, and consequently promoting an increase of the abundance of the phenol-degrading microorganisms or possibly their specific conversion rate (Eq. S6).

Finally, regarding to the fourth mechanism considered (i.e., an increase in intermediate compounds involved in the conversion of phenolics), it could be possible that an increase in the HCO_3_^–^ concentration, due to the fermentation of acetate, could have played a role in the enhancement of the sPhCR by promoting the carboxylation step needed for phenol degradation. It has been reported that in the absence of HCO_3_/CO_2_ phenol degradation under anaerobic conditions is hampered (Karlsson et al., 1999) and that the phenylphosphate carboxylase found in *Syntrophorhabdus* sp. is highly dependent on HCO_3_/CO_2_ (Karlsson et al., 1999; Schühle and Fuchs, 2004; Nobu et al., 2015). However, this hypothesis should be further tested.

## Conclusion

The present study showed the feasibility of using AnMBR for the treatment of phenolic wastewater at high sodium concentrations. From the conducted experiments, performed with previously acclimated biomass, the following conclusions can be derived:

• In batch reactors at 8 gNa^+^⋅L^–1^, phenol at a concentration of 0.5 g⋅L^–1^ decreased the SMA with 27% in comparison to the control tests without phenol. A maximum sPhCR of 17.8 ± 2.6 mgPh⋅gVSS^–1^ d^–1^ was determined for an initial phenol concentration of 500 mg⋅L^–1^.
• In the AnMBR, a stable sPhCR of 40 mgPh⋅gVSS^–1^ L^–1^ was measured when phenol contributed to 80% of the total COD. However, the sPhCR could not be maintained when phenol was the sole CES.
• In the AnMBR, when acetate was added as additional CES, a maximum sPhCR of 115 mgPh⋅gVSS^–1^ L^–1^ was determined.
• The highest sPhCR of 200 mgPh⋅gVSS^–1^ L^–1^ was found when a 2:1 acetate-butyrate mixture, based on COD, was fed to the AnMBR.
• During the operation of the reactors, the most abundant microorganisms were the phenol degrader *Syntrophorhabdus* sp. and the acetoclastic methanogen *Methanosaeta* sp., making more than 50% of the microbial community. Seemingly, there was a correlation (*p* < 0.05) between the increase in the sPhLR and the increase in the relative abundance of *Syntrophorhabdus* sp.

## Data Availability Statement

The original contributions presented in the study are publicly available. This data can be found in NCBI under accession number PRJNA663299.

## Author Contributions

VG conceptualized and designed the batch and reactor experiments, carried out the batch experiments, gathered, and analyzed the data, directed and supervised the operation of reactors, analyzed their data, planned and worked the samples for the microbial community analysis, and wrote the manuscript. JM conceptualized and designed the batch and reactor experiments, critically reviewed and provided feedback for the data analysis. LF designed and operated partially the reactor experiments, carried out analytical methods, and analyzed the data. KQ operated partially the reactors and performed laboratory analyses. DC-G did the bioinformatics analyses and performed the microbial community analysis. JM, HS, and JvL provided feedback that helped shape the research, experiments, and data analysis, and critically revised the manuscript. All authors have read and approved the final manuscript.

## Conflict of Interest

The authors declare that the research was conducted in the absence of any commercial or financial relationships that could be construed as a potential conflict of interest.
