# Supplementary material for: Enhancing Phenol Conversion Rates in Saline Anaerobic Membrane Bioreactor Using Acetate and Butyrate as Additional Carbon and Energy Sources
Source: Front Microbiol. 2020 Nov 30;11:604173. doi: 10.3389/fmicb.2020.604173 (PMC7733923; doi:10.3389/fmicb.2020.604173)
Supplement: Supplementary file 1 [file Data_Sheet_1.pdf]

Supplementary Material

S1. ADM1-based equations for phenol degradation

Based on the anaerobic digestion model No. 1 (ADM1) (Batstone et al., 2002) the following set of equations can be proposed to define the growth of phenol degraders and the consumption of phenol:

$$\frac{dX_{Ph}}{dt} = \mu_{Ph} \cdot X_{Ph} - k_{dPh} \cdot X_{Ph}$$ *Equation S1.*

$$\frac{dS_{Ph}}{dt} = -\frac{dX_{Ph}}{dt} \cdot \frac{1}{Y_{Ph}}$$ *Equation S2.*

Where:

X_{Ph} = Concentration of the active phenol degrader population [$gCOD_{VSS} \cdot L^{-1}$].

μ_{Ph} = Growth rate of the phenol degrader population [h^{-1}].

k_{dPh} = Decay rate of the phenol degrader population [h^{-1}].

S_{Ph} = Phenol concentration [$gCOD_{phenol} \cdot L^{-1}$].

Y_{Ph} = Yield of phenol degraders on phenol [$gCOD_{VSS}/gCOD_{phenol}$].

The growth rate of the phenol degraders is considered to follow a Haldane kinetic (Suidan et al., 1988) defined by:

$$\mu_{Ph} = \mu_{maxPh} \cdot \frac{S_{Ph}}{S_{Ph} + k_{sPh} + \frac{S^2}{k_{iPh}}}$$ *Equation S3.*

Where:

μ_{maxPh}= Maximum growth rate of the phenol degrader population [h^{-1}].

k_{sPh}= Half saturation coefficient [$gCOD_{phenol} \cdot L^{-1}$].

k_{iPh} = Haldane inhibition constant [$gCOD_{phenol} \cdot L^{-1}$].

And considering that μ_{max} could be defined as:

$$\mu_{maxPh} = k_{maxPh} \cdot Y_{Ph}$$ *Equation S4.*

Where:

k_{maxPh} =Maximum specific uptake rate of the active phenol degrader population [$gCOD_{phenol} \cdot gCOD_{VSS}^{-1} \cdot d^{-1}$].

We can substitute in Eq. 4 in Eq. 3

$$\mu_{Ph} = k_{maxPh} \cdot Y_{Ph} \cdot \frac{S_{Ph}}{S_{Ph}+k_{sPh}+\frac{S^2}{K_{iPh}}}$$ *Equation S5.*

Substituting Eq. 5 in equation 1.

$$\frac{dX_{Ph}}{dt} = k_{maxPh} \cdot Y_{Ph} \cdot \frac{S_{Ph}}{S_{Ph}+k_{sPh}+\frac{S^2}{K_{iPh}}} \cdot X_{Ph} - k_{dphenol}$$ *Equation S6.*

Substituting Eq. 6 in Eq. 2 and taking into consideration that kd does not imply phenol consumption

$$\frac{dS_{Ph}}{dt} = -k_{maxPh} \cdot \frac{S_{Ph}}{S_{Ph}+k_{sPh}+\frac{S^2}{K_{iPh}}} \cdot X_{Ph}$$ *Equation S7.*

According to equation S6 and S7, the active phenol-degrader population [X_{Ph}] is capable of only use phenol [S_{Ph}] as CES, while other physiological microbial populations use the other CES (Batstone et al., 2002) for example, the acetate degraders [X_{Ac}].

According to equation 7, a higher phenol degradation rate could be promoted by the increase of the active concentration of the phenol degraders [X_{Ph}], an increase in the specific uptake rate [k_{maxPh}] or by the increase in both.

S2. Table S1. SMA inhibition data

Experiment	*Phenol [mg·L⁻¹]*	*Control SMA*	*Condition SMA values*			*Average SMA*	*SMA factor relative to control*
1	50	0.25	0.25	0.27		0.26	1.03
2	200	0.19	0.26	0.22	0.22	0.23	1.27
3	200	0.49	0.38	0.44	0.41	0.41	0.82
4	500	0.40	0.26	0.33	0.32	0.30	0.75
5	500	0.40	0.230	0.26	0.28	0.28	0.71

All SMA values are presented as gCOD-CH_4·gVSS^{-1}d^{-1}

S3. Inhibition of the acetoclastic SMA of the AnMBR sludge by acetate and butyrate degradation activity

The effect of high concentration of acetate on the specific methanogenic activity (SMA) of the sludge was determined with a series of bath test. Even though the maximum acetate COD concentration in the influent of the AnMBRs was set on 2.0 g·L^{-1}, we wanted to confirm that the biomass would not be inhibited by the acetate.

To perform the experiments, 250 mL Scott glass reactors were used. Biomass form the AnMBR was taken to have a final VSS concentration in the batch reactors of 4 g·L^{-1} (I/S = 2 for the control with 2.0 gAc^{-}COD·L^{-1}). Macro- and micronutrient solutions, phosphate solution A and phosphate solution B, and Na^{+} as NaCl were dosed as in the same concentration as detailed for the AnMBRs (Section 2.3). A shaker (New Brunswick^{TM}, Eppendorf, Germany) at 130 rpm and at 35 °C was used for the incubation. Methane production was continuously measured by an AMPTS system (Bioprocess Control, Sweden) following manufacturer's instruction.

To assess the biomass capacity for butyrate degradation, the batch experiments were repeated using as sodium butyrate at 0,5, 2, and 3 gCOD·L^{-1} as the only carbon and energy source.

S3.1 Results and discussion

The batch tests assays, showed that with an initial COD concentration of 10 g·L^{-1}, the biomass had a similar SMA (0.31 ± 0.03 gCOD-CH_4·gVSS^{-1}d^{-1}) value when compared to an initial acetate concentration of 2 gCOD·L^{-1} (0.28 ± 0.14 gCOD-CH_4·gVSS^{-1}d^{-1}) meaning that the acetoclastic methanogenic population in the AnMBR biomass was active and not inhibited even at high acetate concentrations. Although acetate degradation under anaerobic conditions is commonly described following a Monod degradation kinetics (Batstone et al., 2002), it has also been reported to follow a Haldane kinetic, in which there is an inhibition at high substrate concentrations (Kus and Wiesmann, 1995;Vavilin and Lokshina, 1996).

Both SMA values are in the range for acetoclastic methanogenesis of phenol-degrader suspended biomass in a saline matrix (Muñoz Sierra et al., 2017). However, the values are lower to those reported for phenol-degrading granular biomass such as 0.64 (Fang et al., 1996), 1.10 (Zhou and Fang, 1997), 0.72 (Tay et al., 2000), 1.02 (Fang and Zhou, 2000), 0.75 and 0.72 (Tay et al., 2001) gCOD-CH_4·gVSS^{-1}d^{-1}. Also, our values are lower in comparison to the reported SMA values for phenol-degrading granular biomass in saline matrix going from 1.0 to 3.1 gCOD-CH_4·gVSS^{-1}d^{-1} (Wang et al., 2017).

For butyrate, and despite a lag phase of 2.5 days, an average SMA value of 0.18 gCOD-CH_4·gVSS^{-1}d^{-1} was found (data not shown). Only one reported vale in the literature of 0.89 gCOD-CH_4·gVSS^{-1}d^{-1} for phenol-degrading biomass (Fang and Zhou, 2000) was found. Which was higher than the value that we obtained (0.18 ± 0.01 gCOD-CH_4·gVSS^{-1}d^{-1}). However, since butyrate was fully converted into CH_4 it indicated that the sludge had butyrate oxidizer microorganisms confirming that butyrate could be used by the sludge as a CES.

S4. Second experiment for the reactor operation towards the usage of phenol as main carbon and energy source and the related microbial community

To double check whether phenol degradation was feasible with phenol as the main/unique carbon and energy source, a second continuous experiment with the AnMBR 1 (R1(S1)) was carried out following a different strategy.

The experiment began after the recovery the biomass following the intoxication by phenol [0.9 $gPh\cdot L^{-1}$]. The same composition and concentrations of micro- and macronutrient solution, buffer solution, and yeast extract were used for the feeding solution. To carry out the experiment, R1 was operated during 100 days, and the operation was divided into three stages (Figure S.1). During stage 1, acetate [0.9 and 0.7 $gCOD\cdot L^{-1}$] and phenol at different concentrations [0.1, 0.2, 0.4, 0.5, and 0.65 $gPh\cdot L^{-1}$]. At day 53 the second stage started, and the acetate concentration in the influent was decreased to 0.07 $gCOD\cdot L^{-1}$. At day 85 the phenol concentration was decreased 0.65 to 0.56 $g\cdot L^{-1}$. In the third stage, at day 89, acetate concentration in the influent was increased to 0.6 $gCOD\cdot L^{-1}$ and at day 92 phenol was decreased to from 0.56 to 0.50 $g\cdot L^{-1}$. After the dosage of acetate, the sPhCR was regained.

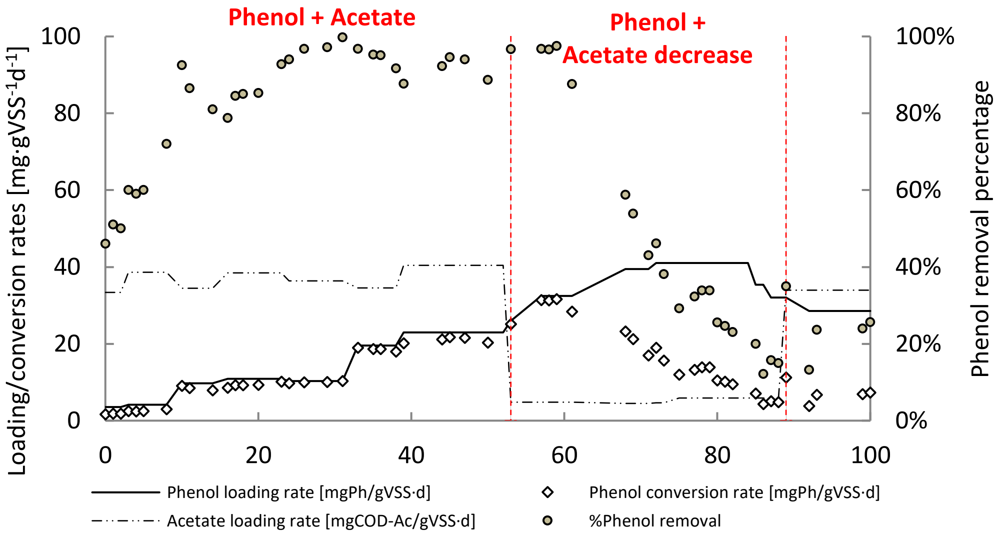

Figure S1. Second operation of the AnMBR towards the usage of phenol as the main carbon and energy source (CaES).

S5. Thermodynamic data for the calculation of ΔG^{01}

Table S2. Thermodynamic data for the different molecules in the phenol degradation reaction

Compound name	***Chemical formula***	***MW (g/mol)***	***Gf^0 (KJ/mol)***	***Reference***
Phenol	C_6H_5OH	94.11	-29.7	Hanselmann *et al.*1991
Acetate	$C_2H_3O_2^-$	59.04	-369.4	Heijnen & Kleerebezem 2010
Butyrate	$C_4H_7O_2^-$	87.09	-352.6	Heijnen & Kleerebezem 2010
Water	H_2O	18.01	-237.18	Heijnen & Kleerebezem 2010
Hydrogen	H_2	2.02	0	Heijnen & Kleerebezem 2010
Proton	H^+	1.01	0	Hanselmann *et al.*1991
electron	e^{-1}	0.00	0	Heijnen & Kleerebezem 2010

S6. Biogas composition and production rate for the AnMBRs operation

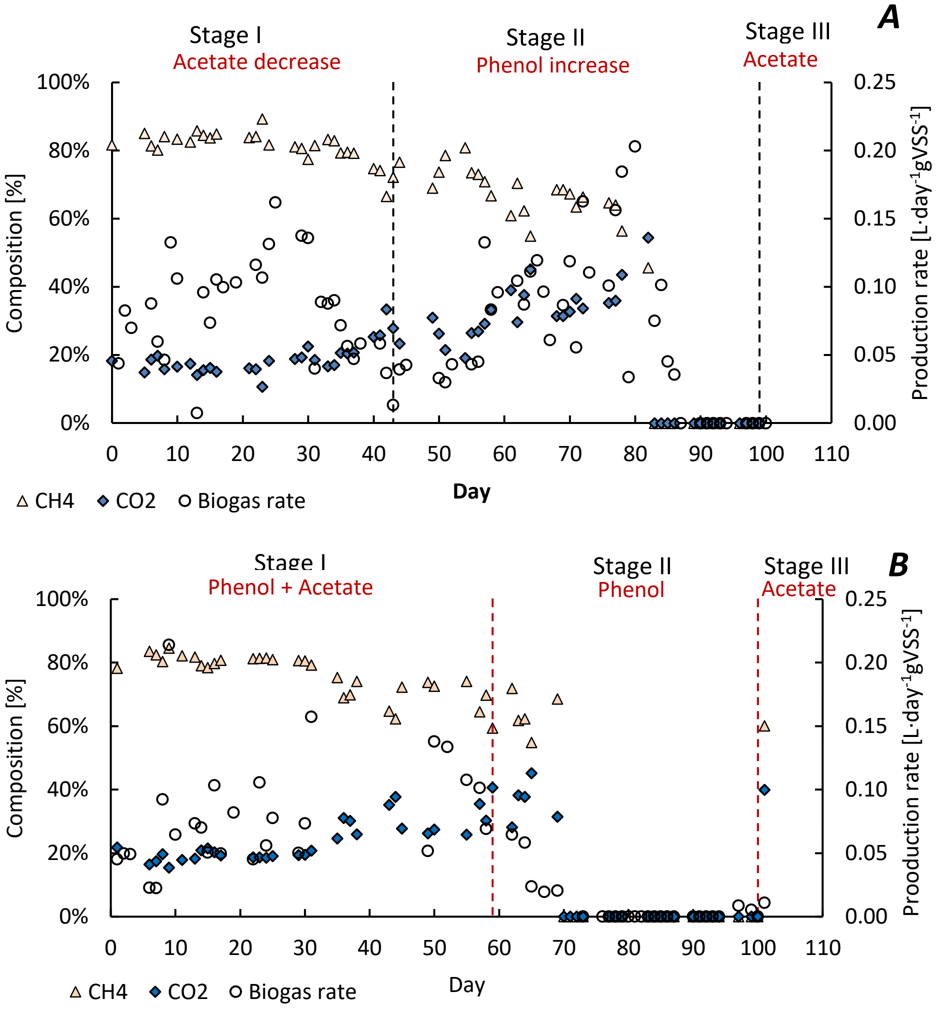

Figure S2. Biogas production rate and composition (CH_4 and CO_2) for R1, and R2(a).

References

Batstone, D.J., Keller, J., Angelidaki, I., Kalyuzhnyi, S.V., Pavlostathis, S.G., Rozzi, A., et al. (2002). The IWA Anaerobic Digestion Model No 1 (ADM1). *Water Science and Technology* 45**,** 65-73 DOI: 10.2166/wst.2002.0292 %J Water Science and Technology.

Fang, H.H., and Zhou, G.-M. (2000). Degradation of phenol and p-cresol in reactors. *Water science and technology* 42**,** 237-244.

Fang, H.H.P., Chen, T., Li, Y.-Y., and Chui, H.-K. (1996). Degradation of phenol in wastewater in an upflow anaerobic sludge blanket reactor. *Water Research* 30**,** 1353-1360 DOI: https://doi.org/10.1016/0043-1354(95)00309-6.

Hanselmann, K.W. (1991). Microbial energetics applied to waste repositories. *Experientia* 47**,** 645-687 DOI: 10.1007/BF01958816.

Heijnen, J.J., and Kleerebezem, R. (2010). "Bioenergetics of Microbial Growth," in *Encyclopedia of Industrial Biotechnology*.), 1-66 DOI: 10.1002/9780470054581.eib084.

Kus, F., and Wiesmann, U. (1995). Degradation kinetics of acetate and propionate by immobilized anaerobic mixed cultures. *Water Research* 29**,** 1437-1443 DOI: https://doi.org/10.1016/0043-1354(94)00285-F.

Muñoz Sierra, J.D., Lafita, C., Gabaldón, C., Spanjers, H., and Van Lier, J.B. (2017). Trace metals supplementation in anaerobic membrane bioreactors treating highly saline phenolic wastewater. *Bioresource Technology* 234**,** 106-114 DOI: http://dx.doi.org/10.1016/j.biortech.2017.03.032.

Suidan, M.T., Najm, I.N., Pfeffer, J.T., and Wang, Y.T. (1988). Anaerobic biodegradation of phenols inhibition kinetics and system stability. *Journal of environmental engineering* 114**,** 1359-1376.

Tay, J.-H., He, Y.-X., and Yan, Y.-G. (2000). Anaerobic biogranulation using phenol as the sole carbon source. *Water environment research* 72**,** 189-194.

Tay, J.-H., He, Y.-X., and Yan, Y.-G. (2001). Improved anaerobic degradation of phenol with supplemental glucose. *Journal of environmental engineering* 127**,** 38-45.

Vavilin, V.A., and Lokshina, L.Y. (1996). Modeling of volatile fatty acids degradation kinetics and evaluation of microorganism activity. *Bioresource Technology* 57**,** 69-80 DOI: https://doi.org/10.1016/0960-8524(96)00052-1.

Wang, W., Wu, B., Pan, S., Yang, K., Hu, Z., and Yuan, S. (2017). Performance robustness of the UASB reactors treating saline phenolic wastewater and analysis of microbial community structure. *Journal of Hazardous Materials* 331**,** 21-27 DOI: https://doi.org/10.1016/j.jhazmat.2017.02.025.

Zhou, G.-M., and Fang, H.H.P. (1997). Co-degradation of phenol and m-cresol in a UASB reactor. *Bioresource Technology* 61**,** 47-52 DOI: http://dx.doi.org/10.1016/S0960-8524(97)84698-6.
